# Are doctors and nurses associated with coverage of essential health services in developing countries? A cross-sectional study

**DOI:** 10.1186/1478-4491-7-27

**Published:** 2009-03-31

**Authors:** Margaret E Kruk, Marta R Prescott, Helen de Pinho, Sandro Galea

**Affiliations:** 1 University of Michigan School of Public Health, Department of Health Management and Policy, Ann Arbor, Michigan, USA; 2 Averting Maternal Death and Disability Program Heilbrunn Department of Population & Family Health, Mailman School of Public Health, Columbia University, New York, New York, USA; 3 University of Michigan School of Public Health, Department of Epidemiology, Ann Arbor, Michigan, USA

## Abstract

**Background:**

There is broad policy consensus that a shortage of doctors and nurses is a key constraint to increasing utilization of essential health services important for achieving the health Millennium Development Goals. However there is limited research on the quantitative links between health workers and service coverage rates. We examined the relationship between doctor and nurse concentrations and utilization rates of five essential health services in developing countries.

**Methods:**

We performed cross-national analyses of low- and middle-income countries by means of ordinary least squares regression with coverage rates of antenatal care, attended delivery, caesarean section, measles immunization, tuberculosis case diagnosis and care for acute respiratory infection as outcomes. Doctor, nurse and aggregate health worker (sum of doctors and nurses) concentrations were the main explanatory variables.

**Results:**

Nurses were associated with utilization of skilled birth attendants (P = 0.02) and doctors were associated with measles immunization rates (P = 0.01) in separate adjusted analyses. Aggregate health workers were associated with the utilization of skilled birth attendants (P < 0.01) and measles immunization (P < 0.01). Doctors, nurses and aggregate health workers were not associated with the remaining four services.

**Conclusion:**

A range of health system and population-level factors aside from health workers influences coverage of health services in developing countries. However, it is also plausible that health workers who are neither doctors nor nurses, such as clinical officers and community health workers, may be providing a substantial proportion of health services. The human resources for health research agenda should be expanded beyond doctors and nurses.

## Background

Attaining the Millennium Development Goals (MDGs) for health, which call for dramatic reductions in child and maternal mortality and halting the spread of HIV/AIDS, malaria and tuberculosis (TB), requires widespread coverage of essential health services [1-3]. While many of the determinants of health lie outside the bounds of the health system, epidemiological and historical evidence support the crucial role of a set of basic health services in reducing child and maternal mortality and the burden of infectious diseases in developing countries [4,5].

There has been a substantial amount of work to define the essential health basket that would be needed to reach the MDGs [3,6]. Reducing child mortality globally requires, for example, the integrated provision of immunization, timely treatment for malaria, diarrhoea and acute respiratory infection as well as components of antenatal care (e.g. tetanus toxoid) [5].

Effective interventions to decrease maternal mortality include assistance from a skilled health professional (doctor, nurse or midwife) at delivery and access to emergency obstetric care, including caesarean section [7]. Reducing the incidence and mitigating the health consequences of infectious diseases requires population and individual level prevention, diagnosis and timely provision of effective treatment [8-10].

Although experts recommend the goal of universal or near-universal coverage of essential health interventions (e.g. > 90% coverage of populations in need) and policy-makers have embraced it, this is far from the current reality in much of the developing world [3,11]. Thus, while virtually every woman in the developed world is assisted by a skilled attendant at delivery, only 32% of women in sub-Saharan Africa receive similar care [7].

Shortages of health workers have been identified as one binding constraint to reaching the high levels of utilization needed to achieve the MDGs [12-15]. As the Joint Learning Initiative (JLI), an expert group on human resources for health, stated: "the only route to reaching the health MDGs is through the [health] worker; there are no short cuts"[16] The JLI estimated that sub-Saharan Africa needs approximately one million more health workers to meet the health MDGs.

While the term "health workers" encompasses a wide range of professionals from doctors to pharmacists to health administrators, much of the literature on health worker shortages has focused on doctors and nurses, in large part because of data availability [17,18]. The causes of health worker shortages in developing countries are multifactorial and include insufficient medical and nursing training, job attrition due to poor wages, job dissatisfaction or ill health, and emigration to wealthier countries [19-21]. For example, Eckhert estimates that all 64 countries of sub-Saharan Africa graduated just 5100 physicians in 2002, compared to 18 000 in the United States [22].

The predilection of doctors and nurses for urban areas in both the developed and developing worlds and with it reduced access to health care for rural populations has been well documented [13,23]. As a result, a growing number of international and national initiatives have aimed to increase the stock of doctors and nurses in developing countries through increasing training, retention incentives and reducing opportunities for emigration [14,21,24,25].

Despite the broad consensus about the central role that health workers play in achieving the health MDGs, there is limited research on the quantitative links between health workers and utilization of health services. Available analyses provide somewhat contradictory evidence on the contributions of different categories of health workers and the role of health workers relative to other health system inputs in increasing utilization of essential services, particularly in developing countries.

At the global level, simple correlations between health worker concentrations (doctors, nurses and midwives per 1000 population) and coverage of essential services suggest that more workers are associated with greater use of some services, including measles vaccination and use of skilled birth attendants [13,21]. Aggregate health workers and doctors alone were associated with measles vaccination coverage in a more recent analysis adjusting for potential confounders such as GDP, female literacy and land area [26]. Two separate analyses also found total health worker density negatively associated with maternal mortality but disagreed about the association with child mortality [17,26].

However, another study found that emigration of doctors and nurses from 53 countries in Africa was not associated with declines in utilization of skilled birth attendants or treatment for infections, suggesting that these two categories of health workers were not independently associated with utilization when controlling for other health system inputs [27]. To our knowledge there are no other analyses in the peer-reviewed literature that have assessed the relation between health workers and a broad range of health services.

In this paper, we investigated the cross-national relation between availability of doctors and nurses and coverage of essential health care services in low- and middle-income countries, which are the focus of the MDGs. To this end, we examined whether concentrations of doctors and nurses/midwives were associated with utilization of six MDG-related essential health services when controlling for other known determinants of utilization. The services were: antenatal care, use of skilled birth attendants, caesarean section, measles immunization, TB case diagnosis and care for acute respiratory infection (ARI).

## Methods

### Variables and data sources

The dataset comprised countries designated as low- or middle-income (2006 gross national income per capita < USD 11 116) by the World Bank for which health service utilization statistics were available in the World Health Organization's (WHO) national health statistics database (WHOSIS) [28]. While there are many essential health services for which there is consensus on inclusion in an essential health package, we selected six health services for our dependent variables; they had to be generally delivered by health workers, have an impact on an MDG health condition, and be reported widely for low- and middle-income countries.

The services selected were: measles immunization, clinic visits of children with acute respiratory infection (presumed pneumonia), antenatal care (minimum four visits), use of skilled birth attendant for delivery, caesarean section and TB case detection under Directly Observed Treatment Short Course (DOTS). All the dependant variables were expressed as the proportion utilizing the service of the population in need, which varied by service. In the case of caesarean section, the variable is expressed as caesarean sections as a percentage of live births. WHO estimates that 5% to 15% of births may require caesarean section due to maternal or fetal complications [29]. These services address MDGs Four (to reduce by two-thirds the under-five mortality rate), Five (to reduce by three-quarters the maternal mortality ratio), and Six (to combat HIV/AIDS, malaria and TB).

Our main independent variables of interest were three health worker measures: concentration of doctors, concentration of nurses and midwives and aggregate concentration of health workers (doctors, nurses and midwives) per 1000 population [17,21,30]. Because of overlapping training and roles and inconsistent reporting of midwife numbers, nurses and midwives were combined in our analysis [26,30]. Health worker data were taken from WHOSIS.

The confounders considered were gross domestic product income per capita (adjusted for purchasing power parity), adult female literacy rate, land area of the country (km^2^), and the proportion of the population living in a rural area. As per Speybroeck et al. [25], income per capita was included to account for a country's overall level of wealth; country wealth, as a proxy for socioeconomic factors, is associated with health services through multiple pathways including health expenditure [17,30-32]. Adult female literacy was included because of the association between education of the mother and use of services as well as the overall increase in the demand of health care and health resources [17,26,33-35].

Consistent with Anand and Bärnighausen [29] and Speybroeck et al. [25], land area was included to account for the logistic difficulties, such as transportation issues, faced by those seeking care as well as health care workers in providing coverage. The proportion of the population living in a rural area, however, was also included to account for different availability of health care infrastructure (e.g. water, sanitation, etc.) that may affect those living or working in rural areas [7,36,37].

These data were collected from the World Bank's World Development Indicators database (WDI) [38]. For countries with missing WDI values for adult female literacy rates, we used female literacy values from the United Nations Educational, Scientific and Cultural Organization [39]. Three countries (Hungary, Poland and Saint Lucia) did not have adult female literacy rates from either source and therefore we used the 2006 *Human development report *for these values [40].

### Statistical analysis

For analysis, the year of the independent variable was matched to the year of the dependent variable. If the exact year value was not available for the independent variable, the closest value reported within five years of the dependent variable was selected (preceding the dependent variable where possible). If data were not available within five years of the dependent variable, the country was eliminated from analysis. In addition, we eliminated nine countries from the caesarean section models where the proportion receiving caesarean section was greater than 15%. A national rate greater than 15% suggests that some caesarean sections may be performed without compelling medical indication, and as such do not represent a life-saving service.

To reflect the boundedness of the dependent variable (all values fall between 0% and 100%), we transformed the health service utilization data using the logistic form. All independent variables were ln-transformed to have the non-linear patterns better fit model assumptions of a linear association between the independent and dependent variables [17,26,30]. We first performed bivariate regressions of health workers and each service. We then performed six multivariate regressions with the full set of independent variables for each of the health services, using separate doctor and nurse concentrations as well as aggregate health workers. To test for the sensitivity of the results to model specification, we also performed multivariate analysis using an arcsin transformation of the dependent variable, as per Speybroeck et al. [26].

## Results

Data for health workers, adult female literacy, GDP, land area and rural population were available for 106 countries (Additional file 1). Information on health services was available for 45 (care for ARI) to 97 countries (use of skilled birth attendants). Table 1 shows descriptive statistics for the variables used in the analysis. Excluding use of caesarean sections, health service utilization varied with lowest coverage for care for ARI (43.7%, standard deviation (SD) 15.0) and highest coverage for measles immunization (83.2%, SD 16.8).

**Table 1:** Descriptive statistics

**Variable**	**N**	**Mean**	**Median**	**STD**	**Min**	**Max**	**Year ranges**
Live births delivered by skilled birth attendant (%)	97	70.9	74.0	26.5	6.0	100.0	1999–2006

Live births delivered by caesarean section (%)	55	5.9	4.0	4.5	0.0	15.0	1998–2006

Children < 1 vaccinated with measles immunization (%)	89	83.2	88.0	16.8	20.0	99.0	2005

Live births preceded by four antenatal care visits (%)	78	61.7	69.0	26.0	10.0	100.0	1999–2006

Case detection rate of tuberculosis under DOTS (%)	81	54.5	57.0	22.9	3.0	100.0	2005–2006

Children < 5 with ARI taken to health care facility (%)	45	43.7	42.8	15.0	11.8	72.6	1999–2006

Physicians per 1000*	106	1.2	0.6	1.3	0.0	5.2	1997–2005

Nurses and midwives per 1000*	106	2.5	1.4	2.6	0.1	12.2	1997–2004

Health care workers per 1000*	106	3.6	2.3	3.7	0.2	16.7	1997–2005

Gross domestic product per capita (PPP)*	106	4715.5	3860.0	3704.2	593.5	15913.0	1998–2005

Adult female literacy* (%)	106	72.5	80.5	25.3	11.9	99.7	2001–2005

Land area (1000 km^2^)*	106	836.2	265.6	2020.4	0.5	16381.4	1998–2005

Population living in rural area (%)	106	51.7	52.1	20.4	6.6	90.0	1998–2005

* Descriptive statistics created from all countries used in any of six health services analyses using most recent statistics

Table 2 shows the results of the bivariate and multivariate regression. The relation between doctors, nurses and aggregate health workers and the independent variables of interest was significant in all bivariate (unadjusted) models with signs in the expected (positive) direction. In the multivariate models, higher doctor concentration was significantly associated with greater use of measles immunization and higher nurse concentration was associated with greater use of skilled birth attendants. Aggregate health worker concentration was positively and significantly associated with use of skilled birth attendants and measles immunization. The adjusted R^2 ^values were highest for utilization of skilled birth attendants (0.60) and caesarean section (0.57), indicating that our set of independent variables explained much more of the variability in these two service coverage rates than the others, for which adjusted R^2 ^values ranged from 0.03 to 0.39 in the models with separate values for doctors and nurses.

**Table 2:** Bivariate and multivariate regression results

	**Antenatal care**	**Use of skilled birth attendant**	**Caesarean section**	**Measles immunization**	**TB case diagnosis**	**Care for respiratory infection**
Bivariate associations	N = 78	N = 97	N = 55	N = 89	N = 81	N = 45

Density of doctors (per 1000)	0.5(< 0.01)	1.4 (< 0.01)	0.4 (< 0.01)	0.6 (< 0.01)	0.2 (< 0.01)	0.2 (< 0.01)

Density of nurses and midwives (per 1000)	0.7(< 0.01)	2.0(< 0.01)	0.6(< 0.01)	0.8(< 0.01)	0.4(0.01)	0.3(< 0.01)

Density of health workers (per 1000)	0.8 (< 0.01)	2.1(< 0.01)	0.6(< 0.01)	0.8(< 0.01)	0.4(0.01)	0.3(< 0.01)

Model set 1 (doctors and nurses)						

GDP per capita (PPP)	0.38 (0.23)	1.00 (< 0.01)	0.58 (0.02)	0.41 (0.07)	0.37 (0.23)	0.14 (0.46)

Female literacy rate (%)	0.92 (0.04)	0.24 (0.68)	0.94 (< 0.01)	-0.23 (0.53)	0.28 (0.54)	0.61 (< 0.01)

Density of doctors (per 1000)	-0.11(0.59)	0.39 (0.14)	-0.03 (0.81)	0.41 (0.01)	-0.05 (0.81)	-0.07 (0.51)

Density of nurses and midwives (per 1000)	0.21 (0.38)	0.75 (0.02)	0.02 (0.89)	0.27 (0.18)	0.11 (0.70)	0.05 (0.69)

Land area (km^2^)	-0.37(< 0.01)	-0.26 (0.02)	-0.03 (0.72)	-0.08 (0.22)	0.01 (0.94)	0.10 (0.16)

Population in rural area (%)	-0.68(0.14)	-0.68 (0.28)	-0.25 (0.60)	0.78 (0.06)	0.01 (0.98)	-0.21 (0.52)

Adjusted R^2^	0.39	0.60	0.57	0.38	0.03	0.34

Model set 2 (aggregate health workers)						

GDP per capita (PPP)	0.29 (0.34)	1.05 (< 0.01)	0.60 (0.01)	0.46 (0.05)	0.37 (0.22)	0.10 (0.56)

Female literacy rate (%)	0.80 (0.07)	0.28 (0.63)	0.92 (< 0.01)	-0.14 (0.69)	0.27 (0.54)	0.58 (< 0.01)

Density of health workers (per 1000)	0.22 (0.37)	1.16 (< 0.01)	-0.003(0.98)	0.69(< 0.01)	0.04 (0.87)	0.01 (0.93)

Land area (km^2^)	-0.36(< 0.01)	-0.25 (0.03)	- 0.03 (0.71)	-0.07 (0.29)	0.004 (0.97)	0.10 (0.13)

Population in rural area (%)	-0.50(0.24)	-0.63 (0.30)	-0.25 (0.60)	0.68 (0.09)	0.04 (0.95)	-0.15 (0.65)

Adjusted R^2^	0.39	0.60	0.58	0.37	0.05	0.35

Note: all independent variables are ln-transformed; all dependent variables are transformed using logistic form

The arcsin-log transformed models using all available countries did not differ substantially from the logit-log models (data available on request).

## Discussion

In cross-national analyses we found that aggregate concentrations of doctors and nurses were associated with utilization of skilled birth attendants and measles immunization but not with four other essential services. In disaggregated analysis, nurses were significantly associated with skilled birth attendant coverage and doctors with measles coverage.

These results are plausible, given known patterns of health service delivery in developing countries. The association between the concentration of nurses and utilization of skilled birth attendants is not surprising, given the definition of skilled birth attendant (doctor, nurse and midwife) and general shortages of physicians in developing countries.

An explanation for the association between physician concentrations and measles immunization is less self-evident, as nurses and other health personnel are generally more involved in delivering vaccines than are doctors. The existing literature on this association is conflicting; while studies using predominantly low-income country data have not found an association between doctors and measles immunization, other work using whole-world country data has found such an association [26,30].

The association we documented may be due to the greater involvement of doctors in the provision of medical care to infants in middle-income countries than in low-income countries. It is possible, however, that this association is an artifact: a result of omitted country-level factors related to both physician density and vaccine rates (e.g. managerial competence of ministries of health and education). Therefore, other factors may be influencing the association we see between doctors and vaccine coverage.

We did not find any associations between doctors and nurses and coverage of the other essential health services: antenatal care, TB diagnosis and care for ARI. There are many possible explanations for this lack of association and we discuss three here in more depth: measurement error, other health system factors that influence coverage rates and finally, the possibility that health workers other than doctors and nurses provide many of these essential services.

Measurement error is a concern in any analysis based on data compiled from several sources (e.g. surveys, national administrative reporting, etc.), such as the WHO data on service coverage used here. While WHO aims to standardize the reporting of coverage rates from different countries, it is possible that the available data are not perfectly comparable. Health worker estimates may also be inaccurate, particularly for nurses. Nurse training and professional designations differ substantially across countries and nurse workforce estimates may not be completely accurate or comparable across countries [17]. While we attempted to limit the amount of measurement error by obtaining data from two sources (WHO and WDI), measurement error is inevitably present and our inferences should be viewed in light of this limitation.

Both health system and other inputs play an important role in increasing coverage of health services, and therefore may be responsible for the lack of association between health care workers and essential services. Some of these factors, such as availability of drugs, supplies, facilities, ambulances, roads and electricity, are at least partly captured by the GDP per capita variable that was significant in two of the analyses (use of skilled birth attendants and caesarean section). Reaching high levels of health service coverage may be more difficult for larger countries with more remote populations, as suggested by the negative associations we found between country size and antenatal care and use of skilled birth attendants [23]. Female literacy increases household demand for health care. Female literacy was significant in our models for caesarean section and care for ARI. However, factors not included here due to data limitations, such as the extent of road and facility infrastructure and donor off-budget funding for health services, may also influence health service coverage. For example, a policy of health user fees has been shown to reduce utilization of a variety of health services [41-43]. Conversely, donor assistance for immunization and tuberculosis programs boosts coverage [44,45]. Moreover, it is possible that as numbers of doctors increase, their focus shifts from essential services to more complex care and therefore coverage of antenatal care and other primary care services does not increase.

Finally, it is possible that the health workers most responsible for providing these services were not in the analysis. Who are they? In many low- and middle-income countries where health worker education rates are low, the answer may be mid-level health workers (e.g. clinical officers, assistant medical officers, nurse technicians) and community health workers [46]. Mid-level health workers or non-physician clinicians – clinicians who generally receive three or more years of medical training after completing secondary school and are delegated tasks traditionally reserved for doctors or nurses – may be particularly important [47].

Many developing countries have been training alternative cadres of health staff since colonial times and, given the chronic shortages of doctors and nurses, continue to rely on these health workers today [46,48,49]. They are active in a wide range of medical activities ranging from child and maternal health care to the diagnosis and treatment of infectious diseases to surgery [47,50-52].

While weak health information systems make it is impossible to estimate their current numbers with any degree of precision, at least in some countries they may provide a bulk of services, particularly in rural areas. A recent review found that these workers were active in 25 of 47 countries across Africa and that in nine countries their numbers exceeded those of doctors [47]. In Mozambique, surgically-trained assistant medical officers performed more than 90% of all major obstetric surgery in rural areas of the country in 2002 [53].

Community health workers, who are community members with basic health training and varying levels of responsibility, may also be involved in providing some of the more basic services. For example, researchers in Southeast Nigeria found that of 252 health workers in 10 primary care clinics, none were doctors, only 8.8% were nurses and the remainder were various cadres of community health workers [54]. Community health workers have also been shown to play an important role in supporting TB and HIV treatment [41,55-58].

While there are no comparable published analyses for the majority of the health services examined here, our findings for use of skilled birth attendants and measles are generally consistent with the work of Speybroeck and colleagues [26]. As already noted, our finding of an association between physician concentrations as well as nurses and midwives and measles differs from the results of a study by Anand and Bärnighausen, likely due to their use of a primarily low-income country dataset [30].

Indirectly, our findings are consistent with those of Clemens, who found that health worker emigration does not affect utilization of basic health services in Africa, when controlling for GDP per capita, education and conflict [27]. He examined the rates of measles and DTP3 vaccination, use of skilled birth attendants, treatment of acute respiratory infection, diarrhoea and HIV and failed to find any effect between doctors abroad per capita and use of those services. He also found that emigration and domestic stock of doctors were significantly and positively associated in adjusted analysis, suggesting that higher numbers of doctors at home do not drive utilization of services.

We did not find a consistent association between the remaining independent variables and the essential health services. Overall, our independent variables predicted the lowest variation of TB case diagnosis and the most in caesarean section. As per Anand and Bärnighausen, income per capita was positively associated with each service but was not always statistically significant; it was not significantly associated with antenatal care, TB case diagnosis and treatment of ARI [29].

For TB this may reflect that TB programmes in many countries are administered and funded through disease-specific mechanisms and are often co-funded by the international community. For treatment of ARI, the lack of association with GDP may reflect the importance of other organizational factors, including quality of medical training and drug supply networks.

For all services except measles immunization, we found a positive association with adult female literacy and use of essential health services. These findings, regardless of statistical significance, were similar to previous studies that found adult female education and literacy were linked with use of and access to essential health services [16,29]. Overall, land area and the fraction of the population that was rural behaved as expected; they were negatively associated with ANC, SBA and caesarean section.

Our analysis had several important limitations. The number of countries with the full set of independent and dependent variables varied for the six services and was relatively small for care for ARI (n = 45). The small samples here mean that the power of our models is low and therefore the inference we can gain from these analyses is limited.

The quality of the health service data that countries report to WHO may vary, particularly when it involves substantial estimation such as the TB case diagnosis (which requires estimation of all smear-positive cases in the country). However, WHO attempts to triangulate the service statistics it receives using multiple data sources.

Because of limited availability of some of the variables, we used the most recent data within five years of the dependent variable. Exact-year matching would have been preferable.

Perhaps most importantly, the data available only permit cross-sectional analyses. Longitudinal work is needed to answer the question of whether increases in physician concentrations will improve utilization of some services or what combination of inputs has the highest potential for improving utilization of essential services.

## Conclusion

Limitations considered, our work suggests that in cross-national comparisons in low- and middle-income countries, concentrations of doctors and nurses are not associated with the differences in provision of several essential health services important for the achievement of the MDGs. While other health system and population factors clearly contribute to higher health care coverage, it is also possible that mid-level and other health providers may be making a substantial contribution to coverage levels of at least some essential services in low- and middle-income countries.

Anecdotal information from developing countries supports the hypothesis that health workers who are neither doctors nor nurses provide a large volume of essential health care, particularly in rural areas. We have some information on who they are – mid-level providers such as clinical officers, assistant medical officers and community health workers – and on their role in a handful of countries [59-62]. However, there remains a large gap in our understanding of these "missing" health workers: how much and what type of care they provide in developing countries, how to ensure that their work is of high quality, and how they can most effectively complement doctors and nurses in expanding access to essential health services.

## Competing interests

The authors declare that they have no competing interests.

## Authors' contributions

MEK, HdP, MRP and SG jointly planned and designed the study. MRP carried out the statistical analysis with oversight from MEK and SG. MK drafted the paper. All authors edited and approved the final manuscript.

## Supplementary Material

Additional file 1 **Full set of countries in analysis.** Click here for file
