# Supplementary material for: Are doctors and nurses associated with coverage of essential health services in developing countries? A cross-sectional study
Source: Hum Resour Health. 2009 Mar 31;7:27. doi: 10.1186/1478-4491-7-27 (PMC2670260; doi:10.1186/1478-4491-7-27)
Supplement: Additional file 1 — Full set of countries in analysis. [file 1478-4491-7-27-S1.doc]

### Additional file 1. Full set of countries in analysis

| **Total country dataset** | **Antenatal care** | **Use of skilled birth attendant** | **Caesarean section** | **Measles immunization** | **TB case diagnosis** | **Care for respiratory infection** |
| --- | --- | --- | --- | --- | --- | --- |
| Albania | X | X | X | X | X |  |
| Algeria | X | X | X | X |  |  |
| Angola |  | X |  | X | X |  |
| Argentina | X |  |  |  |  |  |
| Armenia | X | X | X | X | X | X |
| Azerbaijan | X | X | X |  |  |  |
| Bangladesh | X | X | X | X | X | X |
| Belarus |  |  |  |  |  |  |
| Benin | X | X | X | X | X | X |
| Bolivia | X | X | X | X | X | X |
| Botswana | X | X |  | X | X |  |
| Brazil |  | X |  | X | X |  |
| Bulgaria |  | X |  | X | X |  |
| Burkina Faso | X | X | X | X | X | X |
| Burundi | X | X |  | X | X |  |
| Cambodia | X | X | X | X | X | X |
| Cameroon | X | X | X | X | X | X |
| Cape Verde | X |  |  | X | X |  |
| Central African Republic |  | X |  | X | X |  |
| Chad | X | X | X | X | X | X |
| Chile |  | X |  | X |  |  |
| China |  | X |  | X | X |  |
| Colombia | X | X |  | X | X | X |
| Comoros |  | X |  | X | X |  |
| Congo | X | X | X | X | X | X |
| Costa Rica | X | X |  | X |  |  |
| Cote d'Ivoire | X | X | X | X | X | X |
| Croatia |  | X | X | X |  |  |
| DRC |  | X |  | X | X |  |
| Dominican Republic | X | X |  | X | X | X |
| Ecuador | X | X |  | X | X |  |
| Egypt | X | X | X | X | X | X |
| El Salvador | X | X |  | X | X |  |
| Equitorial Guinea | X | X |  |  |  |  |
| Ethiopia | X | X | X | X | X | X |
| Gabon | X | X | X | X | X | X |
| Ghana | X | X | X | X | X | X |
| Guatemala | X | X | X |  |  | X |
| Guinea | X | X | X | X | X | X |
| Haiti | X |  | X |  |  | X |
| Honduras | X |  | X | X | X | X |
| Hungary |  | X |  | X | X |  |
| India | X | X | X | X | X |  |
| Indonesia | X | X | X | X | X | X |
| Iran | X | X |  | X | X |  |
| Jamaica | X | X |  |  |  |  |
| Jordan | X | X |  | X | X | X |
| Kazakhstan | X |  | X |  |  | X |
| Kenya | X | X | X | X | X | X |
| Latvia |  | X |  | X | X |  |
| Lesotho | X | X | X | X | X | X |
| Lithuania |  | X | X | X | X |  |
| Macedonia |  | X | X | X | X |  |
| Madagascar | X | X | X | X | X | X |
| Malawi | X |  | X |  |  |  |
| Malaysia |  | X |  | X | X |  |
| Mali | X | X | X | X | X | X |
| Mauritania | X | X | X | X | X | X |
| Mauritius |  | X |  | X | X |  |
| Mexico | X | X |  | X |  |  |
| Moldova | X | X | X | X | X | X |
| Mongolia | X | X | X | X | X |  |
| Morocco | X | X | X | X | X | X |
| Namibia | X | X |  | X | X | X |
| Nepal | X | X | X | X | X | X |
| Nicaragua | X | X | X | X | X | X |
| Niger | X | X | X | X | X | X |
| Nigeria | X | X | X | X | X | X |
| Pakistan |  | X |  | X | X |  |
| Panama | X | X |  | X |  |  |
| Papua New Guinea | X | X |  | X | X |  |
| Paraguay | X | X |  | X | X |  |
| Peru | X | X | X |  |  | X |
| Philippines | X | X | X | X | X | X |
| Poland |  | X |  | X | X |  |
| Romania | X | X | X | X | X |  |
| Russia |  | X | X | X | X |  |
| Rwanda | X | X | X | X | X | X |
| Saint Lucia | X | X |  |  |  |  |
| Samoa |  | X |  |  |  |  |
| Sao Tome and Principe |  | X |  | X |  |  |
| Senegal | X | X | X | X | X | X |
| Seychelles |  |  |  | X | X |  |
| Sierra Leone | X | X |  | X | X |  |
| Slovakia |  | X |  | X | X |  |
| Sri Lanka | X | X |  | X | X |  |
| Sudan | X | X |  | X | X |  |
| Suriname | X | X |  | X |  |  |
| Swaziland |  | X |  | X | X |  |
| Syria | X | X |  | X | X |  |
| Tajikistan |  | X | X | X | X |  |
| Thailand | X | X |  | X | X |  |
| Togo |  | X |  | X | X |  |
| Tunisia | X | X | X | X | X |  |
| Turkey | X | X |  | X | X | X |
| Turkmenistan | X | X | X |  |  |  |
| Tanzania | X | X | X | X | X | X |
| Uganda | X | X | X | X | X | X |
| Ukraine |  | X | X | X | X |  |
| Uruguay | X |  |  |  |  |  |
| Uzbekistan |  | X |  | X | X |  |
| Venezuela | X | X |  |  |  |  |
| Viet Nam | X | X | X |  |  | X |
| Yemen | X | X |  | X | X |  |
| Zambia | X | X | X |  |  | X |
| Zimbabwe | X | X | X | X | X | X |
